# Purification and enzymatic characterization of a novel metalloprotease from *Lachesis muta rhombeata* snake venom

**DOI:** 10.1186/s40409-018-0171-x

**Published:** 2018-11-22

**Authors:** Francielle Almeida Cordeiro, Bárbara Marques Coutinho, Gisele Adriano Wiezel, Karla de Castro Figueiredo Bordon, Cristiane Bregge-Silva, Nathalia Gonsales Rosa-Garzon, Hamilton Cabral, Beatrix Ueberheide, Eliane Candiani Arantes

**Affiliations:** 1 Department of Physics and Chemistry, School of Pharmaceutical Sciences of Ribeirão Preto, University of São Paulo (USP), Av. do Café s/n°, Monte Alegre, Ribeirão Preto, SP 14040-903 Brazil; 2 Universidad Latina de Costa Rica, San José, Costa Rica; 3 Department of Pharmaceutical Sciences, School of Pharmaceutical Sciences of Ribeirão Preto, University of São Paulo (USP), Ribeirão Preto, SP Brazil; 4 Proteomics Resource Center, New York University Langone Medical Center, 430 East 29th St, New York City, 10016 USA

**Keywords:** *Lachesis muta rhombeata*, Metalloprotease, Proteases, Snake venom

## Abstract

**Background:**

*Lachesis muta rhombeata* (Lmr) is the largest venomous snake in Latin America and its venom contains mainly enzymatic components, such as serine and metalloproteases, L-amino acid oxidase and phospholipases A_2_. Metalloproteases comprise a large group of zinc-dependent proteases that cleave basement membrane components such as fibronectin, laminin and collagen type IV. These enzymes are responsible for local and systemic changes, including haemorrhage, myonecrosis and inflammation. This study aimed the isolation and enzymatic characterization of the first metalloprotease (Lmr-MP) from Lmr venom (LmrV).

**Methods and results:**

Lmr-MP was purified through two chromatographic steps and submitted to enzymatic characterization. It showed proteolytic activity on azocasein with maximum activity at pH 7.0–9.0. It was inhibited by EDTA (a metal chelator that removes zinc, which is essential for enzymatic activity) and no effect was observed with PMSF, iodoacetic acid or pepstatin (inhibitors of serine, cysteine and aspartyl proteases, respectively). Ca^2+^, Mg^2+^ and Ba^2+^ ions increased its activity, while Al^3+^, Cu^2+^, Ni^2+^ and Zn^2+^ inhibited it. Additionally, ZnCl_2_ showed a dose dependent inhibition of the enzyme. Lmr-MP activity was also evaluated upon chromogenic substrates for plasma kallikrein (S-2302), plasmin and streptokinase-activated plasminogen (S-2251) and Factor Xa (S-2222) showing the highest activity on S-2302. The activity in different solutions (5 mM or 50 mM ammonium bicarbonate, pH 7.8; 0.1% trifluoroacetic acid + 50% acetonitrile; phosphate buffer saline, pH 7.4; 50 mM sodium acetate, pH 4.0 or ammonium acetate pH 4.5) was also evaluated and the results showed that its activity was abolished at acidic pHs. Its molecular mass (22,858 Da) was determined by MALDI-TOF and about 90% of its primary structure was verified by high-resolution mass spectrometry using HCD and ETD fragmentations and database search against the sequence of closely related species. It is a novel enzyme which shared high identity with other snake venom metalloproteases (svMPs) belonging to the P-I group.

**Conclusion:**

The purification procedure achieved a novel pure highly active metalloprotease from LmrV. This new molecule can help to understand the metalloproteases mechanisms of action, the *Lachesis* envenoming, as well as to open new perspectives for its use as therapeutic tools.

## Background

Brazil is one of the countries with the highest number of accidents caused by terrestrial animals, such as scorpions, spiders, snakes, bees and caterpillars wherein more than 54% of them are due to snakes bites [1]. In 2016, the number of accidents caused by snakes in Brazil was 26,295 (under review) and the most dangerous snake genera are *Bothrops*, *Crotalus* and *Lachesis*, the latter representing around 3% of accidents [2]. However, in Northern Brazil the *Lachesis* accidents reach up to 9% [1]. Although the number of accidents is lower than those caused by *Bothrops* and *Crotalus*, the *Lachesis* bites cause a severe envenoming with hypotension, bleeding, pain and a vagal syndrome with diarrhoea, nausea and vertigo [3–5].

*Lachesis* genus, known as “bushmasters”, are the largest snakes in Latin America and the only pit vipers in Brazil that lay eggs [6, 7]. They are currently classified in *Lachesis stenophrys*, *Lachesis melanocephala* (Central America), *Lachesis acrochorda* and *Lachesis muta* (South America). *L. muta* is found in Brazil and subdivided into two subspecies: *L. muta muta* (Amazon tropical forest) and *L. muta rhombeata* (Atlantic forest) [5, 6, 8].

Among the components identified in *Lachesis muta rhombeata* venom (LmrV), are a hyaluronidase [9], phospholipases A_2_ (PLA_2_) [10, 11], phospholipase B (PLB) [9], L-aminoacid oxidase (LAAO) [12], serine protease [9] and bradykinin-potentiating peptides (BPPs) [13]. Although two snake venom metalloproteases (SVMPs) have been identified in *L. muta muta* venom (LmmV), this is the first study with metalloprotease from LmrV.

Metalloproteases are one of the most abundant toxins in Viperid venoms. They are zinc-dependent proteases that cleaves the extracellular matrix (collagen, laminin and fibronectin) and can cause blood coagulation disorders. As consequence, they can lead to haemorrhage, fibrinogenolytic activity, activation of factor X and platelet aggregation inhibition [14, 15].

SVMPs were initially classified into P-I to P-IV classes, however, Fox and Serrano [16] proposed that P-IV class should be inserted into P-III. Therefore, SVMPs were currently classified into P-I, P-IIa and PIIb, and P-IIIa to P-IIId. P-I MPs have 20–30 kDa and contain only the metalloprotease catalytic domain; P-II MPs present 30–60 kDa with protease and disintegrin domains and P-III MPs are within the range of 60–100 kDa with protease, disintegrin and cysteine-rich domain sites [14, 16].

SVMPs have been studied for several therapeutic purposes. Since they interact with cell membrane components, these enzymes have been shown to inhibit angiogenesis, cell migration and adhesion, which are important mechanisms in cancer proliferation and makes these enzymes important tools in metastatic tumours treatment [17–20]. Metalloproteases also can act as therapeutic tools in arthritis disorders [21, 22] and in haemostatic diseases [23].

In this study, we isolated the first metalloprotease from *Lachesis muta rhombeata* venom (Lmr-MP) and its enzyme activity was characterized against azocasein, under different ion concentrations and with the substrates plasma kallikrein (S-2302), plasmin, streptokinase-activated plasminogen (S-2251) and Factor Xa (S-2222). Furthermore, we determined the optimal pH and the mass spectrometry analysis revealed that the glycosylation site observed in other snake metalloproteases is absent in Lmr-MP. The discovery of this new molecule can help to elucidate some mechanisms of action in *Lachesis* envenoming, as well as to contribute to treatment improvement and development of a therapeutic tool for haemostatic diseases.

## Methods

### *Lachesis muta rhombeata* venom

The LmrV was acquired from the Serpentarium Bosque da Saúde in Americana (22° 44′ 21“ S, 47° 19’ 53” W) – São Paulo – Brazil (IBAMA authorization: 647.998). The venom was collected, desiccated and stored at − 20 °C until use.

### Isolation

The crude LmrV (around 23 mg) was dispersed in 500 μL of 0.05 M sodium acetate buffer with 0.15 M NaCl, pH 6.0, centrifuged at 13,400 *xg* at 4 °C for 10 min and the supernatant was filtered on a HiPrep Sephacryl® S-100 HR column (1.6 × 60 cm, GE Healthcare, Sweden) under a flow rate of 0.5 mL/min.

The LmS-6 fraction obtained from the previous step was dispersed in buffer A (0.05 M MES - 2-(*N*-morpholino)ethanesulfonic acid, pH 6.0) and submitted to an ion exchange chromatrography on a HiTrap™ IEX SP XL column (0.7 × 2.5 cm, 1 mL, GE Healthcare). The elution followed a concentration gradient from 0 to 1.0 M NaCl in the same buffer under a flow rate of 0.5 mL/min. The isolation was performed in FPLC Äkta Purifier UPC 900 system with monitoring of 280 nm.

### SDS-page

Sodium dodecyl sulfate polyacrylamide gel electrophoresis (SDS-PAGE) was performed according to Laemmli [24]. A resolution gel containing 13.5% (m/v) bisacrylamide/acrylamide, 1 M Tris-HCl buffer and 0.1% sodium dodecyl sulfate (SDS) was prepared. The concentration gel was prepared with 5% acrylamide in 0.5 M Tris-HCl buffer and 0.1% SDS. The SDS-PAGE was performed under reducing conditions. The SDS-PAGE gel was performed to monitor the isolation process and sample migration was compared to molecular mass standards (Sigma M3913 and M0671).

### Mass spectrometry analysis by MALDI-TOF

The molecular mass of Lmr-MP was determined by MALDI-TOF (Matrix Assisted Laser Desorption/Ionization - Time of Flight) in an Ultraflex II (Bruker Daltonics - DE) mass spectrometer, with a laser source of Nd SmartBeam-YAG laser type (MLN 202, LTB). The solution (1 μL) containing Lmr-MP (5 μg) was spotted with sinapinic acid (SA) matrix (10 mg/mL in a solution containing 0.2% trifluoracetic acid and 80% acetonitrile), in the proportion of 1:1 (*V*/V). Ions were detected using a linear positive mode and calibrated with protein standards from Bruker Daltonics.

### Amino acid sequence determination

**Fig. 1:**
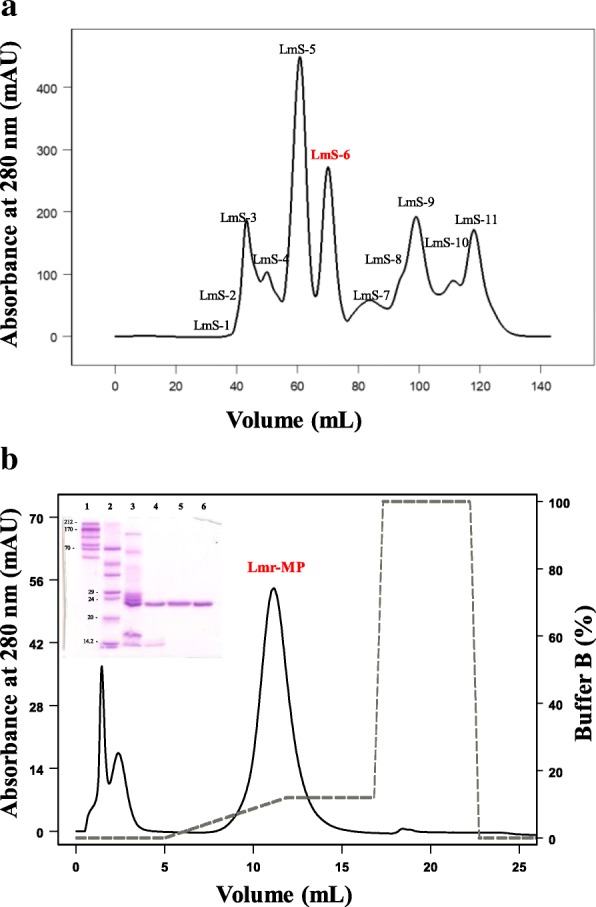
Chromatographic profiles of metalloprotease from *L. m. rhombeata* venom. **a** Fractionation of *Lachesis muta rhombeata* venom by molecular exclusion chromatography on a HiPrep Sephacryl® S-100 HR column (1.6 × 60 cm) using 0.05 M sodium acetate buffer with 0.15 M NaCl, pH 6.0. *L. m. rhombeata venom* (23 mg) was dispersed in 500 μL of buffer. Flow rate: 0.5 mL/min; fractions collected: 1.5 mL/tube. **b** Fractionation of LmS-6 on the ion exchange column HiTrap™ IEX SP XL (0.7 × 2.5 cm, 1 mL). The sample dispersed in buffer A (0.05 M MES - 2-(*N*-morpholino) ethanesulfonic acid, pH 6.0) was applied on the column and eluted using a concentration gradient from 0 to 1.0 M NaCl in the same buffer. Insert SDS-PAGE (13.5%) under reducing conditions. Lanes 1: molecular mass standard (Sigma cat. M0671); 2: molecular mass standard (GE Healthcare 17–0615-01); 3: *L. m. rhombeata* venom; 4: Fraction LmrS-6 from molecular exclusion fractionation; 5 and 6: Lmr-MP

The metalloprotease amino acid sequence characterization was performed with lane 5 from SDS-PAGE (Fig. 1b insert). The gel band was destained with a solution containing 100 mM ammonium bicarbonate (AMBIC: MetOH (50:50) and dehydrated with 100% acetonitrile (ACN). After this, sample was reduced with 100 μL of 1,4-dithiothreitol (3 mg/1000 μL 100 mM AMBIC) for 1 h at 57 °C and alkylated with 100 μL of iodoacetamide (9 mg/1000 μL 100 mM AMBIC) for 45 min, at room temperature and within a dark compartment. For digestion, 222 ng of modified trypsin (Promega™, USA) in 160 μL of 100 mM AMBIC was added and sample was incubated at 25 °C, overnight.

The digested peptides were submitted to an EASY-Spray PepSwift Monolithic Capillary column (Thermo Scientific™) with an Easy-nLC 1000 (Thermo Scientific™) coupled to an Orbitrap Elite™ Mass Spectrometer (Thermo Scientific™, USA). The tryptic peptides were eluted in 65 min using a gradient of ACN from 2 to 90% in 0.5% acetic acid and two independent runs were made. In the first run, the MS spectra were obtained with resolution of 60,000 (at m/z 400) and automatic gain control (AGC) target of 1e6. Subsequently, twenty data-dependent HCD MS/MS were acquired with a resolution of 15,000 (at m/z 400), AGC target of 5e4, normalized collision energy of 35, and isolation window of ±2 Da. In the second run, the MS resolution were obtained with resolution of 120,000 and automatic gain control (AGC) target of 5e4. Twenty data-dependent ETD MS/MS were acquired with a resolution of 15,000 (at m/z 400), AGC target of 5e4, activation time of 60 ms, and isolation window of ±3 Da.

Data from both runs were searched by the software Byonic™ [25] against a database downloaded from UniProt using the keywords “metalloproteinase” and “*Lachesis*” (accession in 07/01/15). Mass tolerance was set as 10.0 ppm for precursor ions and 20.0 ppm for their fragments. Cysteine carbamidomethylation was set as fixed modifications whereas variable modifications included oxidation of methione, N-terminal pyro-glutamate, amidated C-terminal, and HexNAc (N-acetylglucosamine) for N-glycosylation. A wildcard feature of ±150 Da was enabled to search for amino acid substitutions in comparison to the database. The identified peptides were manually checked, specially those different from the database entries.

### Lmr-MP activity in the presence of different inhibitors

The protease class was determined by activity assay in the presence of different inhibitors: ethylene-diaminetetraacetic acid (EDTA), iodoacetic acid (IAA), pepstatin (PEPS) and phenyl-methylsulfonyl fluoride (PMSF). Lmr-MP 100 μL (0.1 mg/mL) was previously incubated at a final concentration of each inhibitor (10 mM) for 5 min at 37 °C [26]. After this previous incubation, 40 μL of HEPES buffer (100 mM, pH 8.0) and 1% azocasein prepared in the same buffer were added. The reaction was performed at 37 °C for 90 min. It was stopped by adding 400 μL of 10% (*w*/*V*) trichloroacetic acid solution. The samples were centrifuged at 10,000×*g* at 25 °C for 15 min and 400 μL of the supernatant were transferred to a new tube and mixed with 467 μL of sodium hydroxide (1 M). The absorbance was measured in a spectrophotometer at 440 nm. One activity unit (U) was defined as the amount of enzyme required to yield an increase of 0.001 A_440nm_ under the assays conditions in according to Morita et al. [27]. The residual activity was determined based on control (without inhibitors) activity: Residual activity = 100 x [(Sample activity)/ (Control activity)].

### Effect of different ions on metalloprotease activity and different ZnCl_2_ concentrations

To determine the effect of different ions on enzyme activity, 100 μL of Lmr-MP (0.1 mg/mL) was previously incubated at a final concentration of 10 mM CoCl_2_, LiCl, MgCl_2_, KCl, ZnCl_2_, NiSO_4_, CuCl_2_, CaCl_2_, MnCl_2_, AlCl_3_, BaCl_2_ and NaCl at 37 °C for 5 min, according to Ducros et al. [28] with modifications. The different ZnCl_2_ concentration (1, 2.5, 5 and 10 mM) effects on Lmr-MP activity was determined after 5 min of incubation at 37 °C. After the previous incubation, 40 μL of HEPES buffer (100 mM, pH 8.0) and 1% azocasein prepared in same buffer were added. The reaction was performed for 90 min at 37 °C and was stopped by adding 400 μL of 10% (*w*/*v*) trichloroacetic acid solution. The samples were centrifuged at 10,000×*g* for 15 min at 25 °C and 400 μL of the supernatant were transferred to a new tube and mixed with 467 μL of 1 M sodium hydroxide. The absorbance was measured in a spectrophotometer at 440 nm. One activity unit (U) was defined as the amount of enzyme required to yield an increase of 0.001 A_440nm_ under the assays conditions in according to Morita et al. [27].

### Lmr-MP activity with different solutions and optimal pH

Lmr-MP activity with 100 μL of different solutions and the optimal pH were performed according to the method of azocasein described in Wang et al. [29]. The sample, fraction LmS-6 (5 μg), was incubated with azocasein (5 mg/mL) in 0.05 M Tris-HCl buffer, pH 8.0, in the presence of different solutions (5 and 50 mM AMBIC pH 7.8; 0.1% TFA + 50% ACN; PBS pH 7.4; 50 mM NaOAc pH 4; 50 mM NH_4_OAc pH 4.5 and 50 mM Tris-HCl pH 8.0) at 37 °C for 90 min. Another assay was carried out with the LmS-6 fraction (5 μg) incubated with azocasein at different pHs (4.5 to 9.0) as described above. The reactions were interrupted with 200 μL of 5% trichloroacetic acid. The mixture was centrifuged at 1000 *xg* for 5 min and 150 μL of each supernatant was transferred to a flat bottom 96-well microplate and added with 150 μL of 0.5 M sodium hydroxide. Albumin (1 mg/mL) was used as negative control and trypsin (1 mg/ mL) as positive control. The absorbance was measured at 450 nm in the Tecan Sunrise Microplate Reader (Tecan, Switzerland).

### Lmr-MP activity upon different substrates

In Chromogenix® chromogenic substrate assay, 5.5 μg of Lmr-S6 was incubated with 200 μL of each substrate (0.04 mM) - plasmatic kallikrein (S-2302), plasmin and plasminogen activated by streptokinase (S-2251) and Factor Xa (S-2222) for 40 min at 37 °C in triplicate. Absorbance reading was performed at 440 nm in the Tecan Sunrise Microplate Reader.

### Statistical analysis

The statistical analysis was performed with the average and standard deviation (SD) and by analysis of variance (ANOVA) with multiple comparisons (Dunnett’s test). The results of *p* < 0.0001, *p* < 0.01 and *p* < 0.05 were considered statistically significant*.* The analyses were performed in triplicate.

## Results

### Lmr-MP isolation

Lmr-MP isolation was performed through two chromatographic steps. Figure 1a shows the first step on a HiPrep Sephacryl® S-100 HR column, in which 11 fractions were obtained and named LmS-1 to LmS-11. LmS-6 showed previously (data not shown) a metalloprotease activity on azocasein and was subsequently submitted to an ion exchange chromatography on HiTrap™ IEX SP XL column (Fig. 1b). In this step, three fractions were obtained. The third fraction was responsible for metalloprotease activity on azocasein (as described below) and was named Lmr-MP.

### SDS-page

Figure 1c insert shows the analysis of the LmrV venom by SDS-PAGE. The crude venom, as well as fraction LmS-6 from the first chromatography step and Lmr-MP isolate in the second chromatographic step, were analyzed. The analysis indicates that Lmr-MP was obtained with high purity and that its molecular mass is approximately 23 kDa, being this mass consistent with metalloproteases belonging to P-I class.

### Mass spectrometry analysis of Lmr-MP

**Fig. 2:**
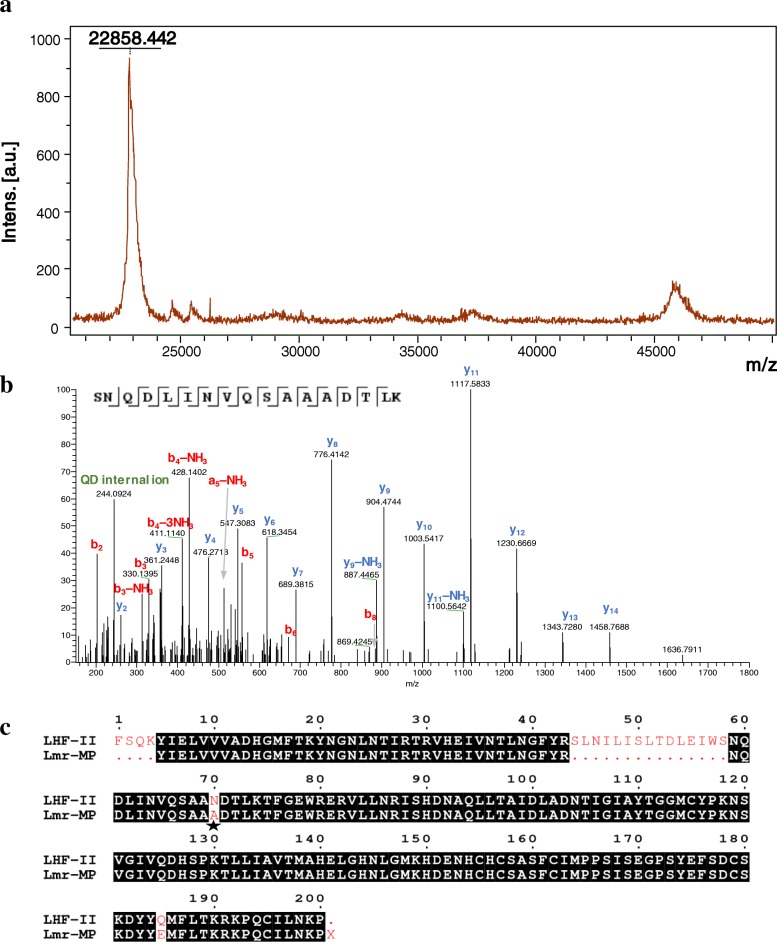
Spectrometry analysis and alignment. **a** Mass spectrum of Lmr-MP. Molecular mass of Lmr-MP was obtained by MALDI-TOF (positive linear mode) using sinapinic acid (SA) matrix. **b** HCD MS/MS of the (M + 2H) ^+ 2^ ion of the tryptic peptide SNQDLINVQSAAADTLK acquired on an Orbitrap Elite™ Mass Spectrometer with 15,000 resolution (at 400 m/z). N-terminal ions (**a** and **b**) are shown in red and indicated by ˩ whereas C-terminal ions (y) are shown in blue and indicated by Г. Internal ions are shown in green. Mass accuracy for all fragment ions is better than 20 ppm. The mass spectrometer used cannot differentiate between leucine and isoleucine residues, and the assignment is made here solely with homology matching. **c** Sequence alignments of sv-MP hemorrhagic factor-2 (LHF-II) from *L. m. muta* (UniProt ID P22796) and Lmr-MP from *L. m. rhombeata*. The highly conserved residues are highlighted in black. Cys residues are shaded gray. Asn-aaX-Ser/Thr residue (star symbol) represents the N-glycosylation site. X = Leu/Ile. Alignment and figure were generated by MultAlin and ESPript servers, respectively

The MALDI-TOF mass spectrometry of Lmr-MP confirmed its high purity level and the molecular mass determined was 22.85 kDa (Fig. 2a). The amino acid sequence analysis was performed on the gel band in lane 5 (Fig. 1 insert). The gel band was trypsinized and Table 1 shows the main peptides identified. The Lmr-MP sequence was compared and aligned with LHF-II, a metalloprotease from *Lachesis muta muta* venom and around 90% of protein sequence was covered (Fig. 2c). The alignment showed that these proteins are very similar although few differences are noted, such as the absence of a potential N-glycosylation site in the 70th position in Lmr-MP in comparison to LHF-II (Fig. 2b and c).

**Table 1:** Tryptic peptides identified through MS/MS analysis

Scan time	Fragmentation mode	MS/MS derived sequence	z	Observed m/z	Calculated m/z	Mass deviation (ppm)	Score
19.56	HCD	TFGEWRER	2	540.7645	540.7647	−0.37	623.7
5.86	HCD	VLLNR	2	307.7031	307.7028	0.97	309.1
28.43	HCD	SNQDLINVQSAA**A**DTLK	2	894.4609	894.4603	−2.35	415.4
35.27	HCD	DYY**E**MFLTK	2	605.2787	605.2785	0.33	288.7
14.12	HCD	YNGNLNTIR	2	532.7779	532.7778	0.19	476.3
10.00	HCD	NSVGIVQDHSPK	2	640.8334	640.8333	0.16	716.8
28.43	HCD	TRVHEIVNTLNGFYR	2	909.9846	909.9841	0.55	730.8
13.42	HCD	KPQCILNKP	2	549.3105	549.3104	0.19	431.7
36.74	ETD	YIELVVVADHGMFTK	3	574.6362	574.6359	0.52	722.1
28.49	ETD	VHEIVNTLNGFYR	3	521.2755	521.2756	−0.19	926.1
3253	ETD	ISHDNAQLLTAIDLADNTIGIAYTGGMCYPK	5	671.3300	671.3296	0.60	1359.6
9.63	ETD	NSVGIVQDHSPK	3	427.5580	427.5580	0	707.5
37.16	ETD	HDENHCHCSASFCIMPPSISEGPSYEFSDCSK	5	755.3034	755.3031	0.40	1248.5
32.92	ETD	TLLIAVTMAHELGHNLGMK	4	517.0287	517.0288	−0.19	1093.1
9.72	ETD	RKPQCILNKP	3	418.5764	418.5764	0	545.7
19.86	ETD	KPQCILNKP**X**	3	404.2375	404.2374	0.25	550.7
18.00	ETD	RKPQCILNKP**X**	3	456.2714	456.2711	0.66	383.5

Cys residues are carbamidomethylated and amino acid residues different from the database are shown in bold. X represents Leu/Ile. M is oxidized Met

### Lmr-MP activity with different inhibitors

**Fig. 3:**
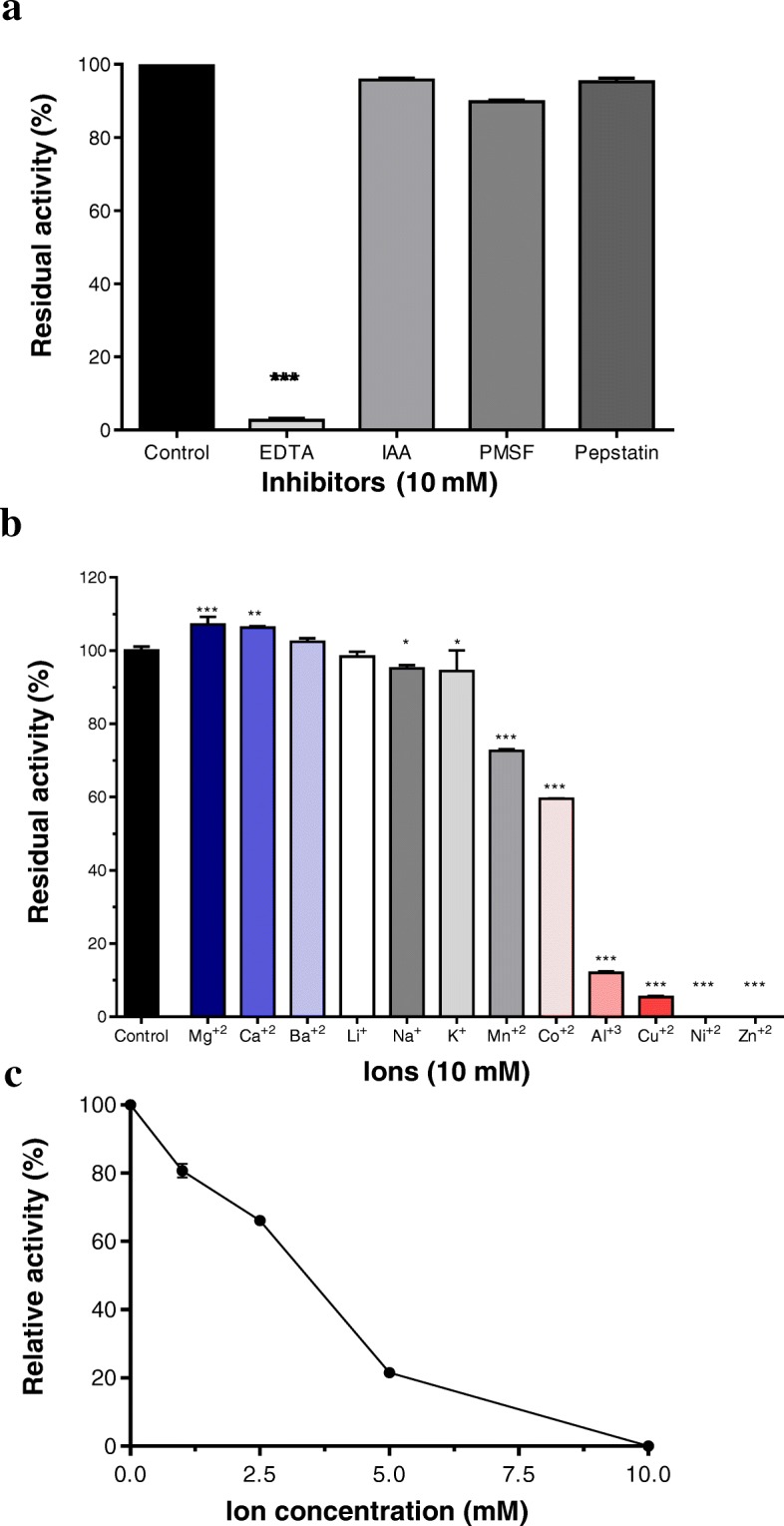
Enzymatic activity of Lmr-MP upon inhibitors and different ions. **a** Azocaseinolytic activity of Lmr-MP (10 μg/100 μL): in the absence (control) or presence of 10 mM different inhibitors (EDTA, IAA, PEPS and PMSF). **b** in the presence of 10 mM different ions (CoCl_2_, LiCl, MgCl_2_, KCl, ZnCl_2_, NiSO_4_, CuCl_2_, CaCl_2_, MnCl_2_, AlCl_3_, BaCl_2_ and NaCl). **c** in the presence of ZnCl_2_ at different concentrations (2.5, 5.0, 7.5 and 10 mM). The reactions were performed at 37 °C. The residual activity was determined based on control activity: Residual activity = 100 x [(Sample activity)/ (Control activity)]. *p < 0.05, ** *p* < 0.01 and *** *p* < 0.0001 compared to the controls (one-way ANOVA, followed by Dunnett’s test). Data (*n* = 3) are presented as mean ± SD

Lmr-MP proteolytic activity was performed with different inhibitors, as EDTA (an ionic chelator that can act as metalloprotease inhibitor), IAA (cysteine protease inhibitor), PMSF (serine protease inhibitor) and pepstatin (aspartyl protease inhibitor). According to Fig. 3a, Lmr-MP activity was completely abolished when incubated with EDTA, suggesting that this enzyme is a metalloprotease, since EDTA is a zinc chelator.

### Effect of different ions and ZnCl_2_ on Lmr-MP activity

Lmr-MP activity was evaluated by enzyme incubation with different ions (CoCl_2_, LiCl, MgCl_2_, KCl, ZnCl_2_, NiSO_4_, CuCl_2_, CaCl_2_, MnCl_2_, AlCl_3_, BaCl_2_ and NaCl). It was observed that Ca^2+^, Mg^2+^ and Ba^2+^ raised the enzyme activity, whereas Al^3+^, Cu^2+^, Ni^2+^ and Zn^2+^ decreased and inhibited the activity (Fig. 3b). Moreover, Zn^2+^, in excess, negatively influences Lmr-MP activity as showed in Figure 3c. Increasing Zn^2+^ concentration decreases Lmr-MP activity.

### LmS-6 activity with different substrate

**Fig. 4:**
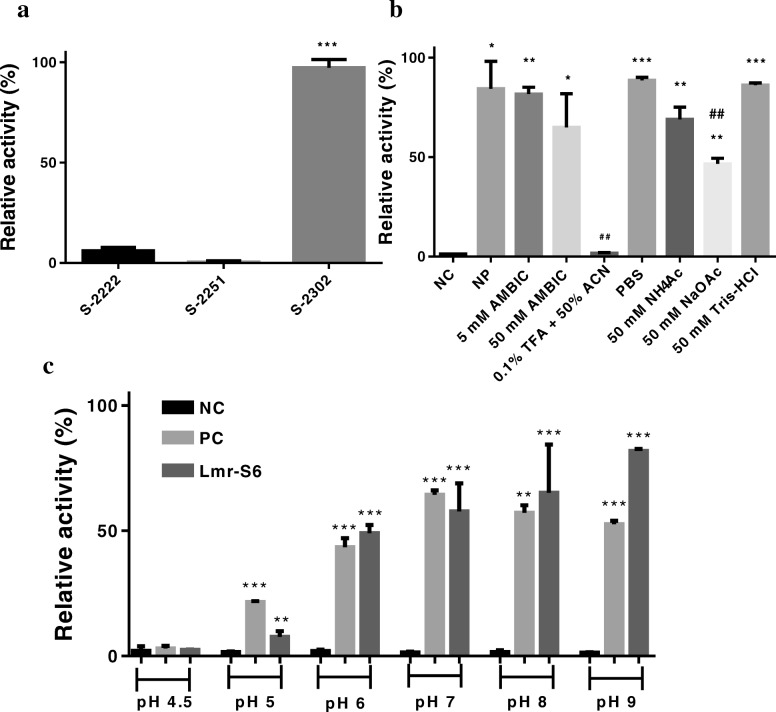
Proteolytic activity of the fraction LmS-6. **a** upon 0.4 mM chromogenic substrates (Chromogenix®) for plasma kallikrein (S-2302), plasmin and plasminogen activated by streptokinase (S-2251) and Factor Xa (S-2222). ***p < 0.0001 compared among the tested substrates. **b** in the presence of different solutions. LmS-6 fraction (5 μg) was incubated with 5 and 50 mM AMBIC, pH 7.8; 0.1% TFA + 50% ACN; PBS, pH 7.4; 50 mM NaOAc, pH 4; 50 mM NH_4_OAc, pH 4.5 and 50 mM Tris-HCl, pH 8.0 at 37 °C for 90 min. Each point represents the mean ± SD (*n* = 3), *p < 0.05, ** *p* < 0.01 and *** *p* < 0.0001 compared to the negative control. The ## symbol represent the significant values when compared to PBS. **c** pH-profile of LmS-6 fraction at different pHs (4.5–9.0). Each point represents the mean ± SD (*n* = 3), *p < 0.05, ** *p* < 0.01 and *** *p* < 0.0001 compared to the controls (one-way ANOVA, followed by Dunnett’s test). NC is negative control and PC positive control

LmS-6 fraction was submitted to chromogenic substrates assay, and the substrate plasma kallikrein (S-2302), H-D-Pro-Phe-Arg-pNA sequence, was cleaved after the arginine residue. The S-2222 substrate with the sequence Bz-Ile-Glu-Gly-Arg-pNA was also cleaved, however with lower affinity. Moreover, the S-2251 substrate was not degraded by LmS-6 (Fig. 4a).

### Lms-6 activity with different solutions and optimal pH

The metalloprotease activity in different solutions and the pH profile were evaluated by azocasein activity with LmS-6 fraction. LmS-6 had the highest activity when incubated with PBS, pH 7.4 and Tris-HCl, pH 8.0, while the enzyme activity was abolished in 0.1% TFA + 50% ACN (Fig. 4b). Moreover, the enzyme was optimally active in a pH range from 7.0 to 9.0 and the activity decreased in acidic pHs (Fig. 4c).

## Discussion

Accidents caused by *Lachesis* genus are less common than *Crotalus* and *Bothrops* envenomings in Brazil. However, large amount of venom is injected in victims bitten by bushmasters leading to severe symptoms such as hypotension, profuse diarrhoea, oedema and abnormal bleeding [3, 30]. These symptoms can be caused by different proteins and peptides present in Lmr venom.

Few components have been purified from *L. muta rhombeata* venom until now, including a L-amino acid oxidase, PLA_2_, PLB, hyaluronidase, serine protease and BPPs [9, 10, 12, 13]. Although there are studies about these components, little is known about some mechanisms induced by these proteins and peptides, especially in *Lachesis* envenomation, since its venom is difficult to obtain and is hard to keep bushmasters in captivity [5, 31].

In this paper we described the isolation of the first metalloprotease from *L. m. rhombeata* subspecies through two chromatographic steps: a molecular exclusion followed by an ion exchange chromatography (Fig. 1). The metalloprotease activity was confirmed by EDTA inhibition on enzymatic assay and no effect caused by other proteases inhibitors (PMSF, IAA and pepstatin) was observed (Fig. 3a). The metalloprotease was named as Lmr-MP and the SDS-PAGE and MALDI-TOF analysis showed that it was efficiently purified (Fig. 1c insert and Fig. 2a). Moreover, the molecular mass determined (22.85 kDa) is in accordance to the mass of other SVMPs and indicates that Lmr-MP is a metalloprotease from P-I class [14, 32, 33].

P-I to P-IIId metalloproteases classification comprehends different multidomains. In P-I case, there is only a signal peptide, a pro-domain and a metalloprotease domain. The signal peptide is responsible for protein secretion, the pro-domain is related to catalytic activation and the metalloprotease domain encodes the enzyme sequence [16, 34]. In general, P-I class metalloproteases are less haemorrhagic than P-III classes possibly because of the multiple domains associated with P-III sequences [32].

Until now, two metalloproteases have been purified from *L. muta muta* venom, LHF-I and LHF-II (also named Mutalysin-II) [35–37]. An alignment between only LHF-II and Lmr-MP was performed since both are P-I SVMPs [38]. The alignment revealed high similarity between these enzymes despite differences in some amino acids (Fig. 2c). LHF-II has a N-glycosylation site in Asn70, while Lmr-MP has an Ala in the same position (Fig 2b). The calculated mass of LHF-II, from its primary sequence, was 22,595.77 Da [39]. In comparison to the molecular mass of Lmr-MP determined by MALDI-TOF, the difference between them is only 262.67 Da, consisted with mass variation regarding amino acid substitutions and not due to glycosylations [40]. Furthermore, the change of Asn by Ala in Lmr-MP lead to the loss of a potential N-glycosylation site.

Pla et al. [41] described the proteome analysis of *Lachesis muta rhombeata* venom. Around 29.5% of venom composition was SVMPs (10.3% of this value are related to metalloproteases from class P-III and 19.2% from P-I class). The authors found similarity of metalloprotease from P-I class with LHF-II described above. In addition, another study from dos Santos [42], also revealed the presence of one metalloprotease from P-I class similar to LHF-II. The metalloprotease found in both studies are probably the Lmr-MP or an isoform.

Although P-I metalloproteases class have no relevant haemorrhagic activity, they degrade other membrane components and appear to be related to pathogenic effects of local damage observed in envenoming [43]. Because of their fibrinolytic and not haemorrhagic effects, the therapeutic potential of these metalloproteases for thrombolytic events has been studied [37], suggesting a potential therapeutic effect for Lmr-MP.

We evaluated Lmr-MP activity in the presence of different ions (Fig. 3b). The enzymatic activity increased with Ca^2+^, Mg^2+^ and Ba^2+^. In contrast, the activity was inhibited by Al^3+^, Cu^2+^, Ni^2+^ and Zn^2+^. Therefore, Lmr-MP is activated by divalent ions. However, zinc ions inhibited this activity. This inhibition by zinc was dose-dependent, as demonstrated in Fig. 3c. Some previous studies have shown that divalent ions, such as Ca^2+^, help to stabilize the protein. On the other hand, although Zn^2+^ is present in metalloprotease structures and is fundamental to proteolytic activity, if in excess, it can inhibit enzyme activity by causing stereochemical disturbances in the stabilization of the molecule [33, 36, 44].

The proteolytic activity of LmS-6 fraction upon chromogenic substrates showed the highest activity when the enzyme was incubated with S- 2302, the substrate for plasma kallikrein (H-D-Pro-Phe-Arg-pNA) (Fig. 4a). Plasma kallikrein is important in human physiology, specifically in release of bradykinin (BK). It is activated by factor XIIa and then cleaves high-molecular-mass kininogen to generate bradykinin [45]. Therefore, the metalloprotease isolated in this study may act in important systems, bringing perspectives for the use of Lmr-MP as a therapeutic agent in haemostatic disorders. Despite this, more studies are needed to prove the activity of the enzyme on the substrate and to infer its activity in envenoming and as therapeutic agent.

Additionally, Lmr-MP activity was evaluated in the presence of different solutions, such as 5 and 50 mM AMBIC (pH 7.8), 0.1% TFA + 50% ACN, PBS pH 7.4, 50 mM NaOAc pH 4, 50 mM NH_4_ OAc pH 4.5 and 50 mM Tris-HCl pH 8.0. The proteolytic activity on azocasein was higher in 50 mM Tris-HCl buffer (pH 8.0) and PBS pH 7.4, which corroborated with optimal pH analysis (Fig. 4c) in which the optimal range was determined between 7.0 and 9.0 (Fig. 4b and c). Moreover, it was observed the loss of metalloprotease activity in 0.1% TFA + 50% ACN solution (Fig. 4b) when compared to the negative control of the assay, probably due to the loss of its cofactor and because of acetonitrile, which may interrupt hydrophobic and ionic interactions [46]. These results also indicate that the metalloprotease loses its activity in acidic pHs. The MP LHF-II is also stable in the pH range from 8 to 10 [36].

## Conclusion

In conclusion, the metalloprotease Lmr-MP of 22,858 Da was efficiently purified from *L. m. rhombeata* venom. Around 90% of Lmr-MP total primary sequence was covered and the MS/MS results altogether with de MALDI-TOF analysis showed that this metalloprotease is not glycosylated (Fig. 2b). The enzymatic assays presented that ion zinc inhibit its activity in a dose-dependent manner and this enzyme lose activity in acidic pHs. These results open new perspectives for *Lachesis* venom and metalloproteases knowledge and, although more studies must be performed to provide a therapeutic activity, Lmr-MP showed preferentially inhibit the kallikrein plasma substrate, that plays a critical role in physiological processes, making it a favorable candidate for future pharmaceutical tools.

## Abbreviations

ACN: Acetonitrile
AlCl_3_: Aluminium chloride
AMBIC: Ammonium bicarbonate
ANOVA: Analysis of variance
BaCl_2_: Barium chloride
BK: Bradykinin
CaCl_2_: Calcium chloride
CoCl_2_: Cobalt chloride
CuCl_2_: Cupric chloride
EDTA: Ethylenediaminetetraacetic acid
ETD: Electron-transfer dissociation
FPLC: *Fast protein liquid chromatography*
HCD: High-energy collisional dissociation
HEPES: 4-(2-hydroxyethyl)-1-piperazineethanesulfonic acid
IAA: Iodoacetic acid
KCl: Potassium chloride
LAAO: L-aminoacid oxidase
LHF-I and II: Metalloproteases from *Lachesis muta muta* venom
LiCl: Litium chloride
Lmr: *Lachesis muta rhombeata*
Lmr-MP: Metalloprotease from *Lachesis muta rhombeata* venom
LmrV: *Lachesis muta rhombeata* venom
LmS-6: Sixth fraction of the *Lachesis muta rhombeata* venom on Sephacryl chromatography
Magnesium chloride: Sodium chloride
MALDI-TOF: Matrix Assisted Laser Desorption Ionisation
MES: 2-(*N*-morpholino) ethanesulfonic acid
MetOH: Methanol
MgCl_2_: Manganese(II) chloride
MnCl_2_: Magnesium chloride
NaOAc: Sodium acetate
NH_4_OAc: Ammonium acetate
NiSO_4_: Nickel sulfate
PBS: Phosphate *buffered* saline
PEPS: Pepstatin
PLA_2_: Phospholipases A_2_
PLB: Phospholipase B
PMSF: Phenylmethylsulfonyl fluoride
SA: Sinapinic acid
SD: Standard deviation SD
SDS-PAGE: Sodium dodecyl sulfate polyacrylamide gel electrophoresis
svMPs: Snake venom metalloproteases
TFA: Trifluoracetic acid
Tris-HCl: Tris hydrochloride buffer
ZnCl_2_: Zinc chloride
